# Altered voxel-mirrored homotopic connectivity in right temporal lobe epilepsy as measured using resting-state fMRI and support vector machine analyses

**DOI:** 10.3389/fpsyt.2022.958294

**Published:** 2022-07-26

**Authors:** Yongqiang Chu, Jun Wu, Du Wang, Junli Huang, Wei Li, Sheng Zhang, Hongwei Ren

**Affiliations:** ^1^Department of Imaging Center, Tianyou Hospital, Affiliated to Wuhan University of Science and Technology, Wuhan, China; ^2^Key Laboratory of Occupational Hazards and Identification, Wuhan University of Science and Technology, Wuhan, China; ^3^Department of Neurosurgery, The Central Hospital of Wuhan, Tongji Medical College, Huazhong University of Science and Technology, Wuhan, China; ^4^Department of Otolaryngology-Head and Neck Surgery, Wuhan Asia General Hospital, Wuhan, China; ^5^Department of Psychiatry, Liyuan Hospital, Tongji Medical College, Huazhong University of Science and Technology, Wuhan, China

**Keywords:** right temporal lobe epilepsy, network homogeneity, voxel-mirrored homotopic connectivity, resting-state functional magnetic resonance imaging, support vector machine analyses

## Abstract

**Background:**

Prior reports revealed abnormalities in voxel-mirrored homotopic connectivity (VMHC) when analyzing neuroimaging data from patients with various psychiatric conditions, including temporal lobe epilepsy (TLE). Whether these VHMC changes can be leveraged to aid in the diagnosis of right TLE (rTLE), however, remains to be established. This study was thus developed to examine abnormal VMHC findings associated with rTLE to determine whether these changes can be used to guide rTLE diagnosis.

**Methods:**

The resultant imaging data of resting-state functional MRI (rs-fMRI) analyses of 59 patients with rTLE and 60 normal control individuals were analyzed using VMHC and support vector machine (SVM) approaches.

**Results:**

Relative to normal controls, patients with rTLE were found to exhibit decreased VMHC values in the bilateral superior and the middle temporal pole (STP and MTP), the bilateral middle and inferior temporal gyri (MTG and ITG), and the bilateral orbital portion of the inferior frontal gyrus (OrbIFG). These patients further exhibited increases in VMHC values in the bilateral precentral gyrus (PreCG), the postcentral gyrus (PoCG), and the supplemental motor area (SMA). The ROC curve of MTG VMHC values showed a great diagnostic efficacy in the diagnosis of rTLE with AUCs, sensitivity, specificity, and optimum cutoff values of 0.819, 0.831, 0.717, and 0.465. These findings highlight the value of the right middle temporal gyrus (rMTG) when differentiating between rTLE and control individuals, with a corresponding SVM analysis yielding respective accuracy, sensitivity, and specificity values of 70.59% (84/119), 78.33% (47/60), and 69.49% (41/59).

**Conclusion:**

In summary, patients with rTLE exhibit various forms of abnormal functional connectivity, and SVM analyses support the potential value of abnormal VMHC values as a neuroimaging biomarker that can aid in the diagnosis of this condition.

## Introduction

Right temporal lobe epilepsy (rTLE) accounts for the majority of partial epilepsy cases (1) and results from excessive abnormal synchronous neuronal firing (2, 3). In prior reports, rTLE has been linked to the deterioration of emotional, cognitive, and psychological functionality with disease progression (4–6). While EEG analyses can be used to diagnose epilepsy, certain patients with TLE experience disease relapse following surgical or medical intervention (7–9). A growing body of evidence also suggests that epileptic activity is linked to the dysfunction of brain networks rather than individual sites in the brain, with these networks contributing to the incidence of interictal brain dysfunction (10).

While many studies explored the development and progression of rTLE with a focus on both clinical symptoms and associated physiological changes (7, 8, 11), the pathogenesis of this condition remains incompletely understood. Different network organization patterns have long been known to be linked to the incidence of left TLE (lTLE) and rTLE owing to hemispheric asymmetry in the human brain (12–14), yet the functional networks associated with lTLE and rTLE differ from one another (10, 14–16). Indeed, one study reported to significantly altered functional connectivity was only evident in lTLE and not in rTLE (17), while another group reported a greater reduction in the functional connectivity of the limbic network in patients with rTLE relative to those with lTLE (18). More prominent reductions in functional connectivity in individuals with rTLE and associated lower baseline levels may suggest that this condition is more amenable to structural and functional changes such that impairment in patients with rTLE may more readily contribute to functional reorganization and lateralization (19, 20). However, data exploring these possibilities have been relatively limited to date, and changes in brain function in individuals with rTLE are not restricted to the temporal lobe, underscoring the need for additional research examining the functional changes in these patients and associated regional involvement.

Progressive advances in neuroimaging techniques have been leveraged to guide the diagnosis of many psychiatric conditions. The change of network homogeneity (NH) is used to explain the aberrant connectivity of the default mode network (DMN) in patients with depression or lTLE (21, 22) and also to explain alterations in the dorsal attachment network (DAN) and the ventral attachment network (VAN) in patients with rTLE (23, 24). Functional connectivity (FC) is often used as a neuroimaging biomarker when assessing intrahemispheric and interhemispheric salience and auditory network abnormalities in patients with somatization disorders (25). Degree centrality (DC) has also been leveraged as an imaging biomarker to aid in rTLE diagnosis (1). Given the growing interest in analyses of interhemispheric connectivity, VMHC has been used to examine functional homotopy between bilateral cerebral hemispheres as a means of comparing interhemispheric FC based on rs-fMRI data, with changes in such connectivity potentially accounting for abnormal homotopic connectivity among specific regions of the brain in patients with rTLE (26). FC has also been leveraged when comparing connectivity strength differences among patients with TLE based upon abnormal VMHC values (27), and VMHC studies revealed altered alertness networks in patients with rTLE (28). This is done by calculating the synergy of functional connections between each voxel in one hemisphere and its mirror voxel in the other hemisphere. As a result, VMHC shows that the pattern of communication between the bilateral cerebral hemispheres is critical for information integration and brain function modifications. Higher VMHC levels indicate better synchronization of functional connections between the brain hemispheres, according to the VMHC data.

The growing complexity of high-dimensional imaging datasets has led to the development of support vector machine (SVM) analyses, which consist of powerful supervised learning models and algorithms that can aid in a variety of classification problems (29). SVM approaches have been successfully used to identify patients affected by autism spectrum disorders, Alzheimer's disease, and cognitive impairment (30–32). However, the utility of VMHC-based SVM analyses as a means of differentiating between patients with rTLE and normal control individuals has yet to be reported.

The present study leveraged rs-fMRI data to compare abnormal functional homotopic connectivity in different regions of the brain in patients with rTLE with the goal of establishing a model capable of differentiating between these patients and healthy control individuals.

## Experimental procedures

### Participants

In total, 59 patients with rTLE diagnosed according to the International League Against Epilepsy diagnostic criteria (33) were recruited from the Tianyou Hospital Affiliated with the Wuhan University of Science and Technology. In addition, 60 age-matched and sex-matched healthy control individuals undergoing standard physical examinations were recruited. To be eligible for study participation, patients with rTLE had to meet the following criteria: (1) patients exhibited standard rTLE symptoms and were diagnosed based on clinical findings, EEG, and MRI analyses; (2) patients were right-handed; (3) patients had undergone routine antiepileptic drug therapy; (4) patients were 18–50 years of age; and (5) patients exhibited a Mini-Mental State Examination (MMSE) score > 24. Patients were excluded if they did not meet any of these criteria, if they had any history of traumatic brain injury, psychiatric disorders, or other neurological conditions, or if they failed to comply with examination procedures or exhibited MRI contraindications. All patients provided written informed consent to participate in this study, which received approval from the Medical Ethics Committee of the Tianyou Hospital Affiliated with the Wuhan University of Science and Technology and was performed as per the Declaration of Helsinki.

### Image acquisition

An Ingenia 3.0 T scanner (Philips, Amsterdam, The Netherlands) was used to collect all rs-fMRI data. Scanning was conducted while patients remained awake and still with their eyes closed using the following settings: repetition time/echo time (TR/TE) 2,000/30 ms; 36 slices; 90° flip angle; 220 mm × 220 mm field of view; 3 mm slice thickness; and 1 mm pitch (34).

### Data preprocessing

MATLAB DPARSF software was used to preprocess data after collection (35). To eliminate the impact of initial signal instability and participant adaptation to the imaging apparatus, the first five-time points were omitted from analyses. Data were corrected for head movement and slice time. No participants exhibited > 2 mm of maximum displacement in the x, y, or z directions or maximum rotation > 2°. The standard Montreal Neurological Institute space was used to normalize the corrected data, which were then resampled at 3 × 3 × 3 mm^3^, subjected to bandpass filtering (0.01–0.08 Hz), and linearly detrended. Covariates of spurious importance were then eliminated, including six head motion parameters derived from rigid body correction and the signal from a region centered in the white matter and the signal from a ventricular seed-based region of interest. Global signal data were retained when analyzing these resting-state results (34).

### VMHC analyses

REST (http://www.restfmri.net/) software was used to conduct all VMHC analyses. Normalized gray matter images for each study participant were averaged together to generate a normalized mean gray matter template. This template was averaged to generate group-specific symmetrical templates, with gray matter images then being registered to the produced symmetrical templates. Each subject's normalized gray matter images were averaged to create a mean nonlinear registration to this symmetrical template, with the associated transformation them being applied to the preprocessed fMRI images for each study participant. Images were then smoothed using a 6 mm full-width at half-maximum isotropic Gaussian kernel. Individual VMHC maps were produced by assessing the Fisher z-transformed Pearson's correlation between a particular voxel and the mirrored voxel in the opposite hemisphere, with the resultant correlations for these voxel pairs being used to establish the VMHC maps utilized for group-level analyses.

### SVM analyses

The MATLAB LIBSVM package was used to conduct SVM analyses aimed at differentiating between patients with rTLE and healthy control individuals based upon VMHC values extracted from the right and left middle temporal gyri (rMTG and lMTG), with this analysis being conducted using a “leave-one-out” approach.

### Statistical analyses

All data were analyzed using SPSS 22.0. Sex distributions were assessed *via* chi-square tests, while age and years of education of patients with rTLE and healthy controls were compared using two-sample *t*-tests. Group differences were identified through an analysis of covariance (ANCOVA) conducted based upon individual whole-brain VHMC maps from the two groups in a voxel-by-voxel manner, with results being GRF corrected at a *p*-value < 0.01.

### Correlation analyses

Mean voxel-based VMHC values were extracted from regions of the brain exhibiting differences between the rTLE and healthy control patient groups, after which Pearson's correlation analyses were used to examine the relationships between these abnormal VMHC values and patient clinical characteristics.

## Results

### Patient characteristics

In total, this study recruited 59 patients with rTLE and 60 healthy controls. No significant differences in age, sex, years of education, or other demographic or clinical characteristics were observed between these groups (Table 1).

**Table 1:** The clinical properties of patients and healthy controls.

**Characteristics**	**Patients (*****n*** = **59)**	**HCs (*****n*** = **60)**	***P*** **value**
Gender (male/female)	59 (27/32)	60 (31/29)	0.519
Age, years	28.97 ± 7.73	26.54 ± 4.96	0.043
Years of education, years	12.37 ± 2.73	12.67 ± 2.33	0.529
Illness duration, years	8.45 ± 6.17		

HCs, healthy controls. Compared with normal controls, *P < 0.01.

### Differences in VMHC values among groups

**Figure 1:**
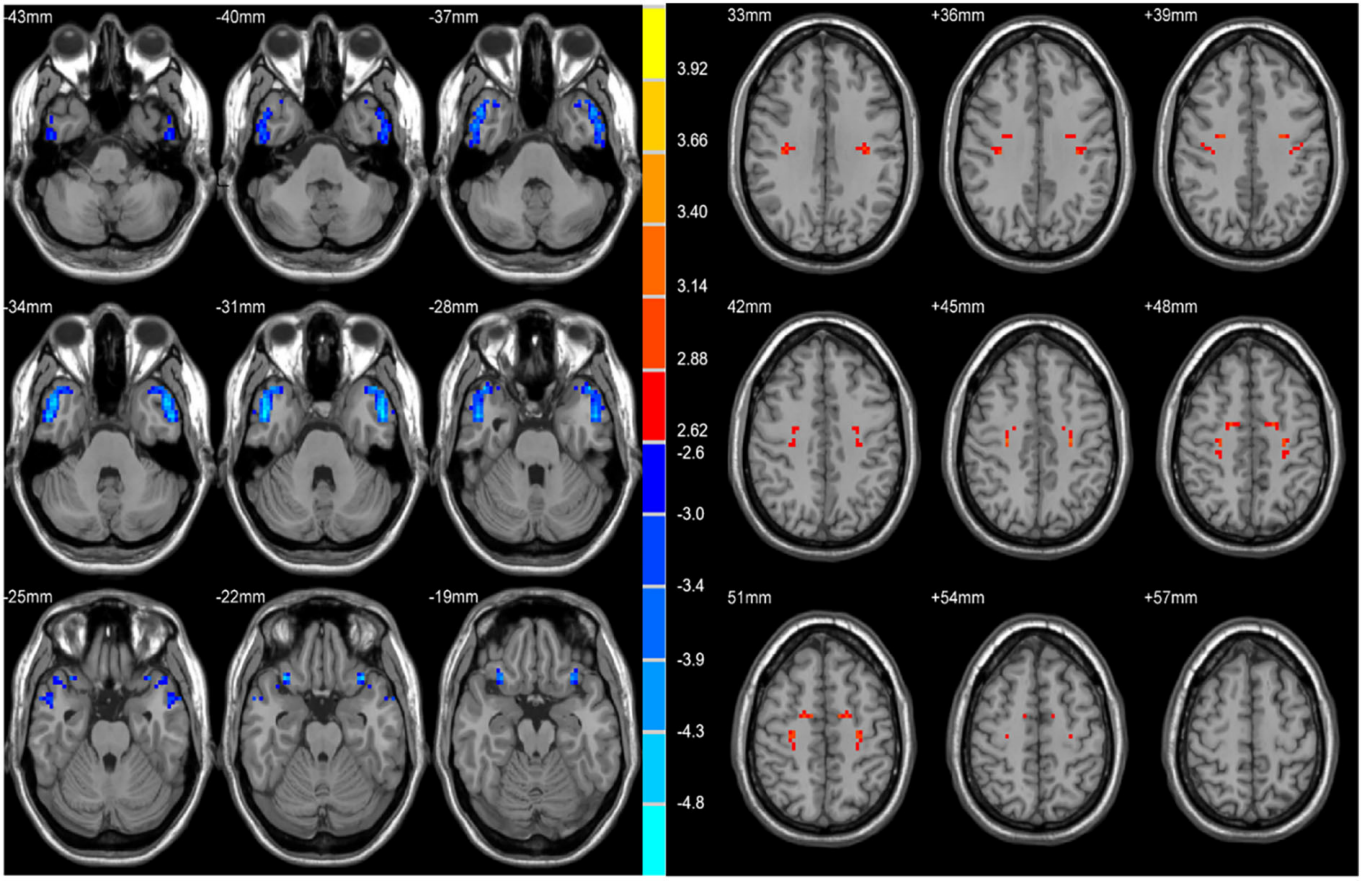
Statistical maps showing VHMC differences between the subject groups. Blue denotes lower VHMC, red denotes higher VHMC, and the color bar indicates the *T* values from 2-sample *t*-tests.

Two-sample *t*-tests revealed significant differences in VMHC values in the bilateral STP, MTP, MTG, ITG, OrbIFG, PreCG, PoCG, and SMA when comparing patients with rTLE to healthy controls (Figure 1 and Table 2). Relative to healthy control individuals, patients with rTLE exhibited a marked decrease in VMHC in the bilateral STP, MTP, MTG, ITG, and OrbIFG, whereas VMHC values in these patients were significantly increased in the bilateral PreCG, PoCG, and SMA.

**Table 2:** Signification differences in VHMC values between the groups.

**Cluster location**	**Peak (MNI)**			**Number of voxels**	***T*** **value**
	**X**	**Y**	**Z**		
MTG	± 51	0	−30	227	−5.2444
PreCG	± 27	−18	45	65	3.1404

*MNI, Montreal Neurological Institute; MTG, middle temporal gyrus; PreCG, precentral gyrus*.

### The ROC curve and SVM results

**Figure 2:**
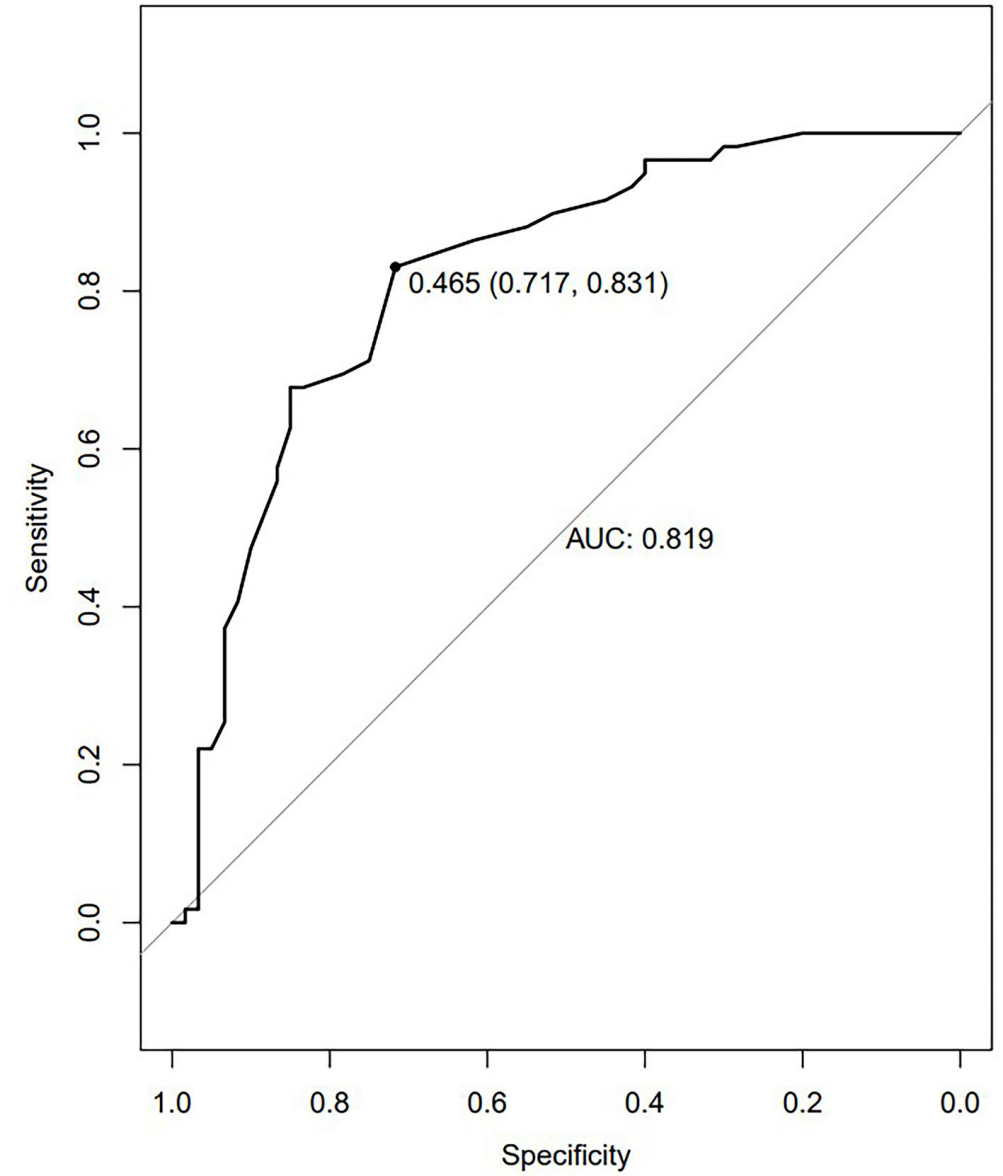
The ROC curves of the altered brain regions in the diagnosis of TLE. The ROC curve of MTG VMHC values. The AUC was 0.819. The optimum cutoff value was 0.465 (sensitivity: 0.831 and specificity: 0.717).

Using VMHC analysis, we confirmed the presence of significant abnormalities in the bilateral MTG (Figure 2). We extracted these two regions as ROIs for the subsequent SVM analysis.

**Figure 3:**
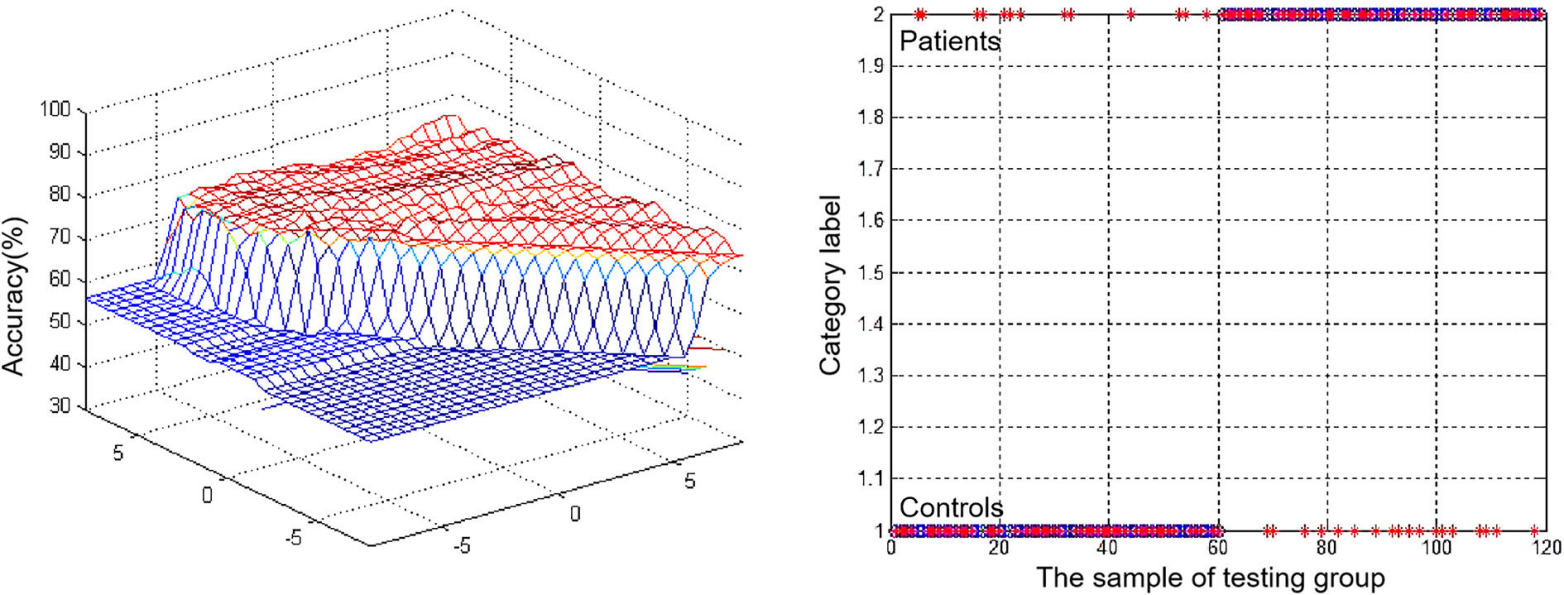
Visualization of classifications through support vector machine (SVM) using the decreased VMHC values in rMTG to discriminate patients with rTLE from healthy controls. Left: SVM parameters result of 3D view. Right: Classified map of the VMHC values in rMTG.

Abnormal VMHC values in the bilateral MTG were separately assessed using an SVM approach, revealing that reduced VMHC values in the rMTG were associated with good diagnostic accuracy, sensitivity, and specificity values of 70.59% (84/119), 78.33% (47/60), and 69.49% (41/59), respectively, when used to differentiate between patients with rTLE and healthy control individuals (Figure 3).

### Correlations between VMHC values and clinical findings

Average VMHC values in four regions of the brain that differed significantly between groups (the bilateral MTG and PreCG) were used to assess any potential correlations between these values and other clinical variables, but no significant correlative relationships were observed in patients with rTLE.

## Discussion

Here, differences in VMHC values in patients with rTLE and healthy control individuals were compared based on whole-brain rs-fMRI data. This approach revealed that individuals diagnosed with rTLE exhibited reductions in VMHC values in the bilateral temporal lobe, particularly in the bilateral MTP and ITG. Moreover, these patients exhibited increased VMHC values in the bilateral PreCG, PoCG, and SMA. SVM analyses further revealed reductions in VMHC values in the rMTG to offer potential value as a neuroimaging biomarker for differentiating between patients with rTLE and healthy control individuals.

The temporal pole (TP) is a structure that has been termed Brain Area 38 by Brodmann or Temporopolar Area TG by Von Economo and that has been suggested to play an integral role in many higher-order cognitive functions (36, 37). The TP has been subdivided into various areas using assorted different methods. For example, diffusion tensor imaging (DTI) connectivity-based research revealed TP afferents from the MTG, ITG, OrbIFG, and the auditory cortex of the superior temporal gyrus in addition to being affected by the superior frontal gyrus (SFG) (38). Other EcoG studies observed TP involvement at seizure onset and posited that this may explain the failure of temporal lobectomy in some cases (7), in addition to confirming the association between the white matter network of the TP and that of other regions. In the present analysis, the TP was defined as the area of the anterior temporal lobe (ATL) beneath the lateral sulcus at the rostral tip of the temporal lobe inside the most rostral portion of the middle cranial fossa. This definition is in line with that used in prior clinical histological and connectivity analyses of the TP (38). Many studies reported the TP to exhibit distinct functions pertaining to autobiographical memory, facial recognition, language and semantic processing, socioemotional processing, and the recognition and analysis of complex objects (36). While many studies confirmed a role for the TP in psychiatric and neurological conditions, as in the case of TP atrophy being related to impaired memory and semantic deficiencies in individuals with Alzheimer's disease, few articles specifically assessed patients with rTLE (39–42). Another analysis of abnormal DMN homogeneity in patients with TLE reported a reduction in network homogeneity in individuals diagnosed with rTLE (34). In this study, patients with rTLE exhibited significantly decreased VMHC values in the bilateral MTP and STP relative to healthy controls, consistent with the existence of homotopic connectivity between the same brain regions as the contralateral side while indicating weaker functional connections with other regions of the brain. It is similar to a study exploring alterations of the alertness network in patients with rTLE, in which reduced FC values were found in STP (43). Aberrant TP brain activity in patients with rTLE and the associated abnormal homotopic connectivity of these regions may thus play an important role in the pathophysiology of rTLE. These results may further help to explain observed decreases in semantic and language processing in patients suffering from this condition.

This analysis also revealed decreased VMHC values in the bilateral ITG and MTG in patients with rTLE. In a previous study assessing alertness in unilateral patients with TLE, similar bilateral reductions in VMHC values in the MTG were reported in study subjects (28). Moreover, resting-state brain entropy analyses in patients with rTLE and the assessment of the relationship between these results, and alertness revealed synchronous alterations in the rMTG and rITG (44). Consistently, another DTI-based study of 67 chronic left hemisphere stroke survivors revealed distinct damage in the ITG and MTG among individuals experiencing word comprehension difficulties, suggesting that these regions play an important role in the integration of auditory and conceptual processing (45). In addition to being linked anatomically by the white matter network, these regions may thus also be linked at a functional level. The results of this study suggest the existence of homotopic connectivity in patients with rTLE between the bilateral MTG and ITG, despite their distinct functions on either side of the brain.

Bilateral OrbIFG VMHC values were also found to be decreased in patients with rTLE, consistent with functional homotopic connectivity and synchronous alterations between the OrbIFG and TL. The arcuate fibers also reciprocally connect the OrbIFG and TP, and these structures make up the uncinate fasciculus (UF) (38, 46). One analysis of a blending aromatic mixture revealed that OrbIFG activation was consistent with a connection with the LP (47), supporting a role for this region in mediating configural percepts between the TP and OrbIFG associated with memory processes. Related studies also reported shifts in language dominance to homologous regions in the other hemisphere of the brain, with DTI having revealed poorer tract integrity in the right UF in patients with rTLE relative to patients with lTLE (19), potentially suggesting better recovery of language function. In our study, VMHC values were also decreased in the bilateral OrbIFG in patients with rTLE, suggesting the existence of tight homotopic connectivity between the TP and OrbIFG. This may explain the observed positive correlations between the OrbIFG and TP in these patients and the rubs in the aspect of the default semantic network (DSN).

In other prior reports, bilateral STP were shown to be positively correlated with SMA (38, 48). There is also a dominant connection between the STP and other major default mode network (DMN) regions, potentially suggesting that the lateral temporal cortex, as a previously defined DMN subsystem, may further extend to the TP (49). DMN has been reported to be associated with the semantic memory system in the functional space (50). The results of this analysis suggest that increased VMHC values in the bilateral SMA may correspond to negative homotopic connectivity between the TP and SMA. One prior electrophysiological analysis demonstrated that SMA stimulation in patients with epilepsy resulted in the elicitation of complex contralateral movements (51). This aligns with the results of the present study indicating increased VMHC values in the left SMA, potential explaining why the abnormal activation of this region can result in certain clinical symptoms such as the promotion of inhibition of movement, thus impacting motor control (52). Given the role that the SMA plays in planning, initiating, and anticipating particular movements, its activation may explain the incidence of abnormal involuntary movements.

It is additionally important to note the increased VMHC values in the bilateral PerCG and PoCG in patients with rTLE, as these regions respectively correspond to the motor and sensory centers and serve as sensorimotor areas (53). In one study of changes in gray matter volume in patients with mTLE, a loss of PoCG volume was found to be negatively correlated with increased EGG current density (11). Another DTI-based study of PerCG and PoCG in the context of normal brain aging revealed that physical training could delay the progression of brain aging with the PerCG as the primary motion cortex (54). An analysis of hand-mouth movements as a representative ethologically relevant behavior exhibited integrated synergies in the PerCG (55). Regional homogeneity analyses in patients with TLP similarly exhibited an increase in the regional homogeneity of the PerCG and PoCG (12). These prior results are consistent with the data from the present study indicating increased VMHC values in these regions corresponding to their abnormal activation. This further suggests that TLE can lead to the abnormal activation of other functional regions of the brain, owing to abnormal connectivity among these regions in the form of a compensatory function.

The advent of increasingly advanced neuroimaging technologies has led to growing interest in the computer-aided diagnosis of neurological diseases and other pathological conditions. SVM approaches have been used with great success in the diagnosis of schizophrenia, Huntington's disease, and major depression (56–60). Here, considering the possible diagnostic role of the altered VMHC values in some different brain regions, the ROC curve was performed, and the results are shown in Figure 2. We found the better AUCs of the ROC were 0.819 for the bilateral MTG with sensitivity and specificity values of 0.831 and 0.717. What can be determined is that VMHC values can be used as a highly accurate diagnostic tool by the reduced VMHC values in bilateral MTG with a cutoff value of 0.465. An SVM analysis of decreased VMHC values in the rMTG exhibited respective accuracy, sensitivity, and specificity values of 70.59%, 78.33%, and 69.49% when differentiating between patients with rTLE and healthy controls. When sensitivity or specificity values were below 60%, the selected biomarker may be poorly suited to use as a diagnostic biomarker (61). This study is the first to our knowledge to have examined the diagnostic utility of abnormal VMHC values in the rMTG as a neuroimaging biomarker of rTLE. In prior reports, DC values have been successfully leveraged as neuroimaging biomarkers in the evaluation of rTLE, with greater accuracy, sensitivity, and specificity being observed when assessing increased DC values in the combination of two abnormal regions of the brain (1). One advantage of this present approach is that abnormal VMHC values in just one region of the brain are necessary to yield comparable diagnostic accuracy, specificity, and sensitivity, making the associated analyses more efficient and intuitive.

There are multiple limitations to this analysis. For one, included patients with rTLE were undergoing long-term treatment with standard antiepileptic drugs, potentially impacting the resultant analyses. Second, the sample size for this study was relatively small. Lastly, further work should be conducted to similarly examine the neuroimaging features capable of specifically aiding in the diagnosis of patients with lTLE.

## Conclusion

In summary, the results of this study suggest that patients with rTLE exhibit several abnormalities in VMHC values consistent with aberrant functional connectivity in the whole brain and among particular brain regions. The SVM results from this study suggest that altered VMHC within the rMTG can be leveraged as a neuroimaging biomarker to differentiate between patients with rTLE and healthy controls. Overall, these findings support the existence of altered bilateral functional coordination in patients with rTLE, offering new insight into the role of functional homotopic dysregulation in this disease and thus providing a foundation for future research aimed at clarifying the underlying pathological basis for rTLE development.

## Data availability statement

The raw data supporting the conclusions of this article will be made available by the authors, without undue reservation.

## Ethics statement

The studies involving human participants were reviewed and approved by the Medical Ethics Committee of the Tianyou Hospital Affiliated to Wuhan University of Science and Technology. The patients/participants provided their written informed consent to participate in this study. Written informed consent was obtained from the individual(s) for the publication of any potentially identifiable images or data included in this article.

## Author contributions

DW, JH, JW, and HR: conceptualization, project planning and methodology, manuscript review, and editing. YC, SZ, and WL: data analysis and manuscript first draft. All authors contributed to the article and approved the submitted version.

## Funding

The investigation was supported by a grant from the Health Commission of Hubei Province Scientific research project (Grant No. 2020CFB512), the Health Commission of Hubei Province Scientific research project (Grant No. WJ2021M007), the Education Department of Hubei Province research project (Grant No. B2021022), and the Natural Science Joint Foundation of Hubei province (Grant No. WJ2019H232 and Grant No. WJ2019H233).

## Conflict of interest

The authors declare that the research was conducted in the absence of any commercial or financial relationships that could be construed as a potential conflict of interest.

## Publisher's note

All claims expressed in this article are solely those of the authors and do not necessarily represent those of their affiliated organizations or those of the publisher, the editors, and the reviewers. Any product that may be evaluated in this article, or claim that may be made by its manufacturer, is not guaranteed or endorsed by the publisher.
